# Analytical Methodology for Trace Determination of Propoxur and Fenitrothion Pesticide Residues by Decanoic Acid Modified Magnetic Nanoparticles

**DOI:** 10.3390/molecules24244621

**Published:** 2019-12-17

**Authors:** Amine Gizem Canlı, Bilge Sürücü, Halil İbrahim Ulusoy, Erkan Yılmaz, Abuzar Kabir, Marcello Locatelli

**Affiliations:** 1 Department of Analytical Chemistry, Faculty of Pharmacy, Cumhuriyet University, 58140 Sivas, Turkey; eczaminegizem@gmail.com (A.G.C.); bilgesurucu@hotmail.com (B.S.); 2 Department of Analytical Chemistry, Faculty of Pharmacy, Erciyes University, 38039 Kayseri, Turkey; erkanyilmaz@erciyes.edu.tr; 3 ERNAM-Erciyes University Nanotechnology Application and Research Center, 38039 Kayseri, Turkey; 4 Department of Chemistry and Biochemistry, International Forensic Research Institute, Florida International University, 11200 SW 8th St, Miami, FL 33199, USA; akabir@fiu.edu; 5 Department of Pharmacy, University of Chieti-Pescara “G. d’Annunzio”, Via dei Vestini 31, 66100 Chieti, Italy

**Keywords:** fenitrothion, propoxur, HPLC, magnetic solid phase extraction, environmental water samples

## Abstract

A sensitive, rapid, reliable, and easily applicable method based on magnetic solid phase extraction (MSPE) combined with HPLC-PDA was developed for monitoring propoxur (PRO) and fenitrothion (FEN) pesticides in environmental water samples. The effect of major experimental variables on the extraction efficiency of both the pesticides was investigated and optimized systematically. For this purpose, a new magnetic material containing decanoic acid on the surface of particles was synthesized and characterized by XRD, FT-IR, SEM, EDX, and TGA analysis in detail. The simultaneous determination of pesticide molecules was carried out by using a Luna Omega C18 column, isocratic elution of acetonitrile (ACN): Water (70:30 *v*/*v*) with a flow rate of 1.2 mL min^−1^. After MSPE, the linear range for pesticide molecules (r^2^ > 0.9982) was obtained in the range of 5–800 and 10–800 ng mL^−1^, respectively. The limit of detections (LOD) are 1.43 and 4.71 ng mL^−1^ for PRO and FEN, respectively while RSDs % are below 3.5%. The applicability of the proposed method in four different environmental samples were also investigated using a standard addition-recovery procedure. Average recoveries at two spiking levels were over the range of 91.3–102.5% with RSD < 5.0% (*n* = 3). The obtained results show that decanoic acid grafted magnetic particles in MSPE combined with HPLC-PDA is a fast and simple method for the determination of PRO and FEN in environmental water samples.

## 1. Introduction

Pesticides are used to increase productivity in agriculture and avoid various insects in residential and other public areas. They can remain on food during the process of growing, collecting, distributing, and consuming vegetables and fruits. Moreover, their residues can exist in the air, water, and soil as the result of transferring these molecules. The pesticides have many adverse effects on humans, environment, and other organisms in the ecosystem. Therefore, analysis of pesticides residues in food and environmental samples is an important challenge and closely monitored by most of the legal agencies in Europe and other developed countries. Therefore, identification and determination of pesticide residues is very critical for the human health and wellbeing, as well as for the ecosystem. When pesticides are applied to an area, some of their molecules evaporate and pass to living things through respiration and the other part condenses on the surface of the soil such as snow, rain, and irrigation water. Then, these substances are consumed by plants or water and accumulate in fat tissues [1].

**Figure 1:**
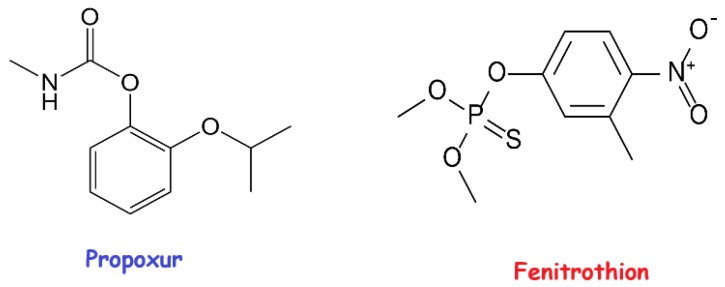
Molecular formulas of the studied pesticides.

As an organic compound, organophosphorus and carbamate pesticides are extensively applied to agricultural areas owing to their great effectiveness, wide spectrum for various species, and low persistence. They are widely used all over the world because they are an attractive alternative to persistent organochlorine pesticides and possess the ability to rapidly degrade under natural conditions sunlight, air, and soil [2]. The carbamate form is a class of highly effective commercial pesticides and they are preferred worldwide since 1960. They are known as N-substituted carbamic acid esters [3]. As a carbamate pesticide, propoxur (PRO) was introduced by German chemical manufacturer Bayer in 1975. It is used against insect pests such as chewing and sucking insects, flies, moths, cockroaches, ants, crickets, and mosquitoes [4]. The toxic properties are based on inhibition of acetyl cholinesterase in the central nervous system, leading to paralysis in insects and mammals [5]. Fenitrothion (FEN) is one of the most common organophosphorus insecticides frequently used to control insects in crops, such as vegetables, rice, fruits, coffee, soybeans, cotton, and cereals. It is also employed to rein the mosquito vector carrying. FEN is partially water-soluble and therefore may enter the ground and underground water [6,7]. Molecular formulas of PRO and FEN pesticides were given in Figure 1.

Contamination of surface water and groundwater with hazardous compounds has attracted increasing attention in recent decades all over the world [8]. According to the European Union (EU) Directive on water quality (98/83/CE) [9], the maximum admissible concentration (MAC) for pesticides is 0.1 μg L^−1^ for each individual substance and 0.5 μg L^−1^ is the maximum allowed for the total concentration of all organophosphorus pesticides. For this reason, development of easy applicable, sensitive, cheap, and correct analysis methods for pesticide molecules is needed as an alternative to the time consuming and expensive chromatographic laboratory techniques currently in use [10]. A lot of analytical method and pretreatment procedures were studied and published for propoxur and fenitrothion pesticides in various samples such as plant and animal tissues, vegetables, fruits, food grains, different type of waters, and milk samples. These methods are generally based on pretreatment procedures or expensive hybrid instrumental tools [3,4,5,11,12,13,14].

Trace analysis of the analytes in complex samples generally requires a pretreatment step to isolate and enrich the target analytes, and to reduce the matrix interferences prior to instrumental determinations [15]. The most common used pretreatment procedure is solid phase extraction (SPE) based on column or batch type because the application is easy and available for automation. Although many sample preparation techniques have been already used by analytical chemists, solid-phase extraction (SPE) based approaches are mostly preferred in routine analytical process because of its versatility and the possibility of using different materials as adsorbent [16,17,18]. There are a variety of sorbents available for various target ions or molecules. However, separation scientists have been trying to develop a better one every day. An appropriate adsorbent is very crucial for the efficient extraction of the analyte; therefore, the exploration of new adsorbents has received growing attention. Among adsorbent materials, carbon materials have attracted substantial interest due to their high surface area, excellent adsorption capacity, good chemical stability, and low cost [19,20]. Magnetic solid-phase extraction (MSPE) is based on magnetic particles as adsorbent that makes the sample preparation procedure simple and fast [21]. In MSPE, target analytes are adsorbed on the surface of magnetic particles and separated from the sample solution by an external magnetic force easily [16,22]. Iron based magnetic particles have significant advantages including primarily price, environmentally safe, high specific surface area, physical and chemical stability, and compatibility for biomedical applications [23]. In addition to these advantageous properties, their most favorable characteristics include easier separation through an external magnetic field, and the absence of internal diffusion resistance [24,25,26]. Moreover, the required selectivity can be obtained by covering the core with various functional groups for target molecules. These are ideal properties expected from an effective preconcentration and separation method [27,28].

The present study reports a simple, rapid, and convenient procedure based on magnetic solid- phase extraction and HPLC-DAD for the trace determination of propoxur and fenitrothion in environmental water samples. The used magnetic particles as solid-phase sorbent was synthetized and characterized for this study. The sensitivity in this MSPE approach is mainly based on synthesized magnetic particle modified by decanoic acid. The finally, the procedure was applied to water samples successfully.

## 2. Results and Discussions

### 2.1. Characterization of Magnetic Nanoparticles

Characterization of the newly developed magnetic material was carried out by FTIR, XRD, SEM-EDX, and TGA analysis. Structure of magnetic sorbent was provided and explained in every step of characterization.

**Figure 2:**
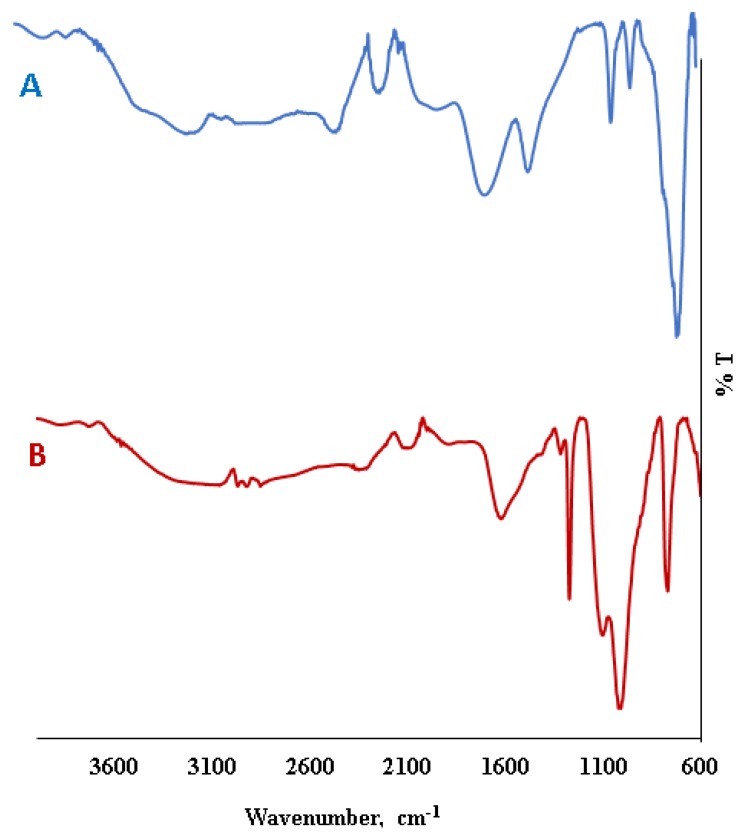
FT-IR spectrum of the Fe_3_O_4_ nanoparticles (**A**) and decanoic acid modified Fe_3_O_4_ nanoparticles (**B**).

Figure 2 illustrates the FT-IR spectrum of the Fe_3_O_4_ and decanoic acid modified Fe_3_O_4_ materials. A specific strong peak around 650 cm^−1^ belongs to Fe-O vibration modes (Figure 2A). When the FT-IR spectrum of decanoic acid modified Fe_3_O_4_ is examined, important shifts in spectrum region and important differences at peak intensities and shapes are seen (Figure 2B). Characteristic peaks at around 1010 and 796 cm^−1^ are corresponding to Si-O-CH_3_ and Si-O vibrations, respectively. The new peaks around 1620 cm^−1^ correspond to C=O vibration in decanoic acid (Figure 2B).

**Figure 3:**
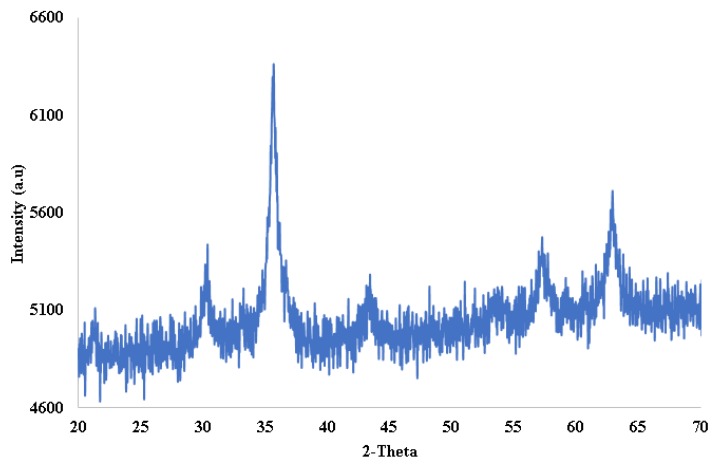
XRD patterns of the decanoic acid modified Fe_3_O_4_ material.

In XRD analysis, Figure 3 shows the XRD patterns of decanoic acid modified Fe_3_O_4_ material. The XRD pattern causes a reflection peak at 2θ = 30.3, 35.7, and 57.2° that can be assigned to Fe_3_O_4_, and 2θ = 62.9° that can be assigned to α-Fe_2_O_3_.

**Figure 4:**
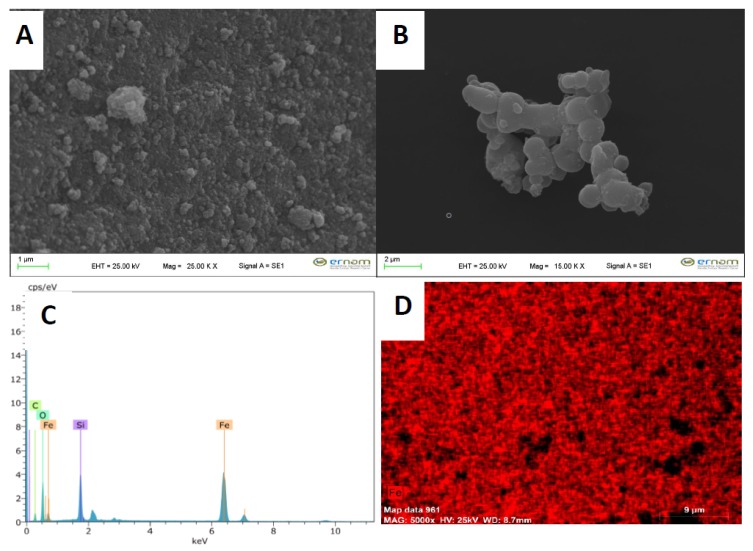
(**A**) Results of FE-SEM analysis of Fe_3_O_4_ nanoparticles, (**B**) decanoic acid modified Fe_3_O_4_ nanoparticles, (**C**) EDX analysis of decanoic acid modified Fe_3_O_4_ nanoparticles, and (**D**) mapping analysis of decanoic acid modified Fe_3_O_4_ nanoparticles.

The morphological structure, particles size of the synthesized Fe_3_O_4_ and decanoic acid modified Fe_3_O_4_ materials were searched by FE-SEM (Field Emission Scanning Electron Microscope), SEM-EDX, and SEM-mapping analysis. The obtained results showed that the average size of Fe_3_O_4_ particles were 108 nm (Figure 4A). After modification of Fe_3_O_4_ particles with tetraethyl orthosilicate and decanoic acid, as well as morphological structure, it was seen that the morphological structure of the material changed completely, and the size of particles increased (Figure 4B). Moreover, formation of Fe_3_O_4_ particles and successful connection of tetraethyl orthosilicate (TEOS) group on the Fe_3_O_4_ particles were proven by SEM-EDX analysis (Figure 4C). Homogeneous dispersion of Fe_3_O_4_ particles in the whole structure was proved by mapping analysis (Figure 4D).

**Figure 5:**
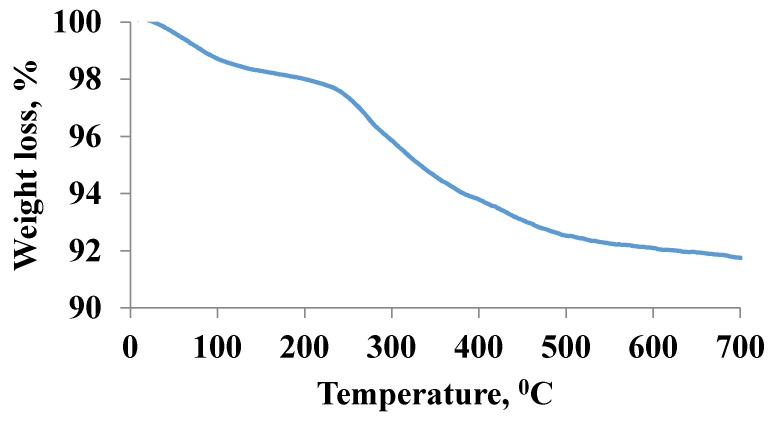
TGA analysis of the decanoic acid modified Fe_3_O_4_ nanoparticles.

In thermo gravimetric analysis, the TGA curve of decanoic acid modified Fe_3_O_4_ is shown in Figure 5. As can be seen in Figure 5, the total weight loss of the material was approximately 8% and the TGA process can be divided into three distinct stages. The first weight loss was at approximately 50–240 °C, corresponding to the evaporation of moisture. The second major weight loss (around 4%) was at approximately 240–400 °C corresponding to conversion of silane group to SiO_2_ and the last weight loss (around 2%) was at approximately 400–700 °C corresponding to decomposition of decanoic acid.

### 2.2. Optimization of Magnetic Solid-Phase Extraction Procedure

Each parameter affecting extraction efficiency of the magnetic nanoparticles was studied and optimized in detail. All optimization steps were studied using model solutions containing 200 ng mL^−1^ propoxur and fenitrothion pesticides. During the optimization of parameters, one parameter is systematically changed while other parameters are kept at constant level. Initial conditions of MSPE procedure: 50 mg of magnetic material, 2 mL of BR buffer, 60 min of adsorption time, 1 mL of organic solvent, 60 s of vortex. All experiments were carried out in 50 mL of falcon tube. The studies have been started at a fixed ratio sample/sorbent (50 mL of sample/50 mg sorbent). Then, all variables were optimized step-by-step by repeating three times (*n* = 3). The analytical signal increased by every step of the optimization and finally arrived at a maximum value for an ideal and sensitive determination of pesticides.

#### 2.2.1. Effect of pH on Extraction Efficiency

**Figure 6:**
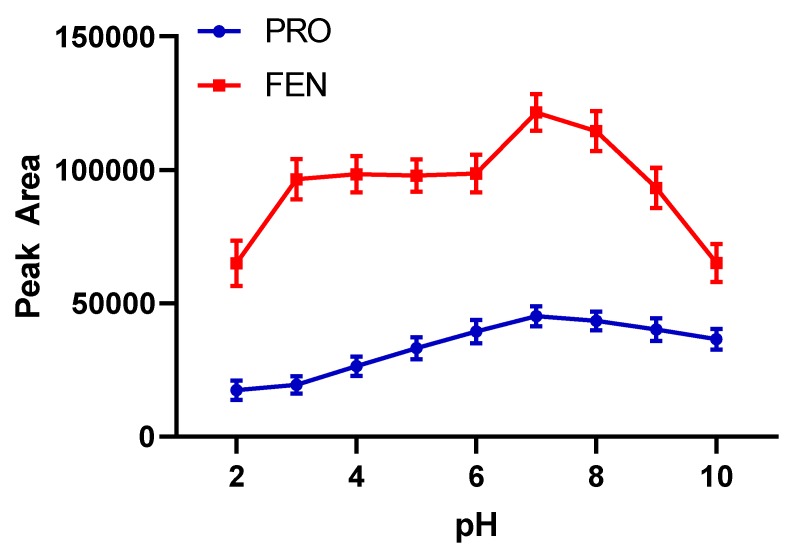
The effect of pH on extraction efficacy (*n* = 3).

Propoxur and fenitrothion pesticides have functional groups affected by the matrix pH. In addition, surface charge of magnetic particles also changes with acidity of the medium. Therefore, an ideal pH value should be determined as the first optimization parameter. The possible major interactions between target molecules and magnetic particles are hydrophobic interactions and π-π stacking. Therefore, most of the analyte molecules are considered in molecular form for a good extraction efficiency. Additionally, carbamate pesticides exhibit the weak alkaline property (pKa values around 12) and would suffer from hydrolysis in strong alkaline conditions, especially when the pH is above 10. The pH of preconcentration medium based on magnetic solid-phase extraction was evaluated in the range of 2–10 by using a BR buffer system. As can be seen in Figure 6, intensity of analytical signals arrived at a maximum value of pH 7.0 for both pesticides. Interactions between analyte and magnetic sorbents are observed in the highest level, so next studies were continued by using pH 7.0 buffers. This value is also good for the application of the method to environmental samples in their own pH which is close to neutral range.

#### 2.2.2. Selection of Desorption Solvent and Its Optimum Volume

**Figure 7:**
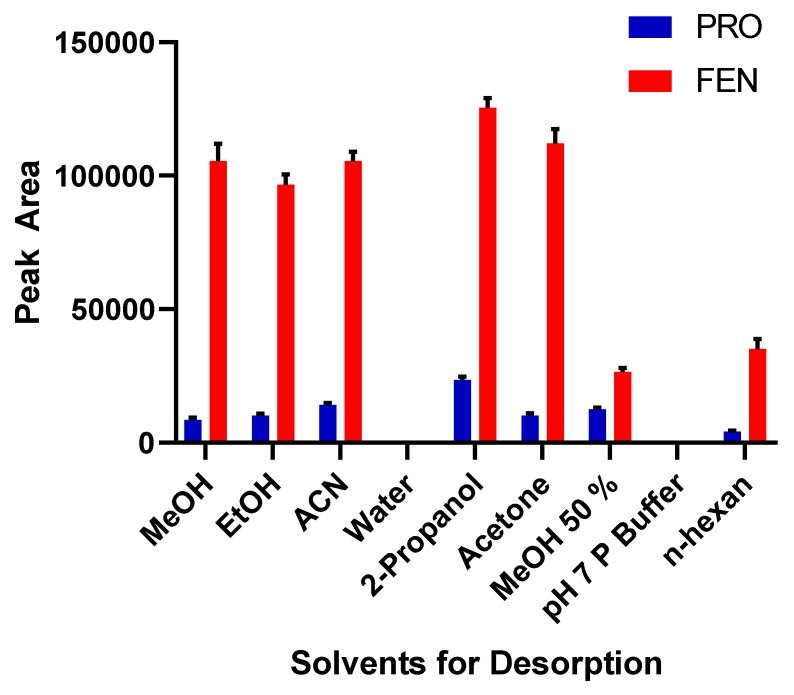
Selection of appropriate desorption solvent (*n* = 3).

The magnetic solid-phase extraction process can be evaluated in two main steps: Adsorption and desorption of target molecules. In the first step, the required conditions for adsorption are enhanced and optimized such as pH, type of magnetic particles, and interaction time. In the second step, the conditions are examined for complete desorption of retained pesticide molecules from the surface of magnetic particles. Desorption is controlled by various mechanisms such as electrostatic interactions and dipole–dipole interactions. In order to find the best suitable solvent for both molecules, polar and nonpolar solvents were studied, and analytical signals were followed for each option. 1 mL of each solvent were used for desorption experiments. The results were shown in Figure 7. Intensity of analytical signals obtaining from 2-propanol for pesticides were higher than other solvents. Therefore, next studies were continued by using this solvent.

**Figure 8:**
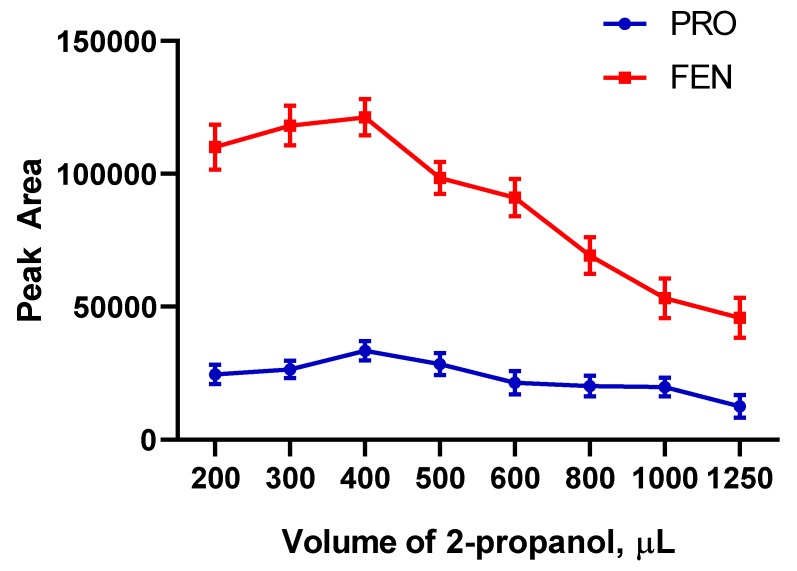
Optimization for volume of desorption solvent (*n* = 3).

The amount of desorption solvent is another important parameter in order to obtain a high preconcentration factor. The final volume of solution determines efficiency of preconcentration procedure because initial volume is the same for all solutions. Therefore, it is aimed at the possible lowest volume of desorption solvent. However, there is another problem in this step about desorption efficiency. If the volume of desorption solvent is not enough to remove all retained molecules from the surface of particles, quantitate results are not obtained in the final step. Therefore, the volume of solvent is crucial in preconcentration experiments. After 2-propanol was chosen as an ideal solvent, its volume was optimized in the range of 200–1300 µL. The results were given in Figure 8. As expected, the analytical signals increased by volume initially and then decreased again due to dilution effect. The peak point for both pesticides was determined as 400 µL of 2-propanol.

#### 2.2.3. Optimization of Shaking Time for Adsorption and Vortexing Time for Desorption

**Figure 9:**
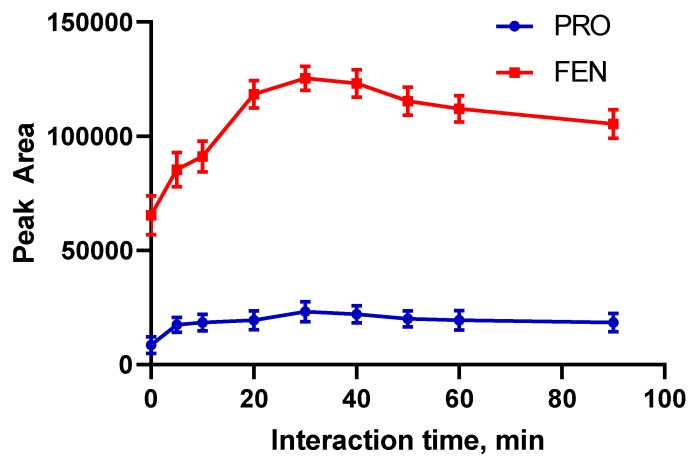
Optimization of adsorption time (*n* = 3).

Retention of pesticide molecules on the surface of magnetic particles needs adequate contact time. For this purpose, an orbital shaker was used at 80 rpm rate. The model solutions containing pesticides were placed on an orbital shaker and interaction time was evaluated in the range of 0–90 min. It is expected that an ideal method should be completed in the shortest time possible. The optimal time should be enough for a quantitate extraction and not much longer for fast analysis. As can be seen in Figure 9, 30 min as interaction time is enough for both pesticides. There is no meaningful change after 30 min. Therefore, the samples were kept on an orbital shaker for 30 min in next studies.

The effect of vortex time in the desorption process was also optimized in the range of 10–120 s. Experiments were repeated by using model solutions including PRO and FEN pesticides at the concentration of 100 ng mL^−1^. Under the optimization conditions, it was observed that 40 s is enough for a quantitate desorption of pesticide molecules.

#### 2.2.4. Reusability of Decanoic Acid Grafted Magnetic Particles

For an ideal solid phase sorbent, the developed materials are expected to offer several attributes such as cost efficiency, resistance against various chemicals, reusability, environmental-friendly, etc. Reusability is one of the most important characteristics of a solid-phase extraction sorbent because it directly impacts on the analysis costs and durability of the method. In order to assess reusability of magnetic particles, experimental studies were repeated with model solutions at the same concentration (involving 100 ng mL^−1^ of each pesticide). As the result revealed, the peak area of pesticides did not change significantly after 10 cycle use. The magnetic particles were washed with 2 mL of 2-propanol and 1 mL methanol after every use. Then, it was dried and re-weighed again. Changes in peak area of pesticides were lower than 5% after 10 uses.

### 2.3. Method Validation

Method validation has been performed as recommended in the International Conference on Harmonization of Technical Requirements for Registration of Pharmaceuticals for Human Use (ICH) [28,29,30].

#### 2.3.1. Linearity

The linearity of the method was studied using twelve spiked levels (in the range of 5–800 and 10–800 ng mL^−1^) for both pesticides and each solution were analyzed by the developed MSPE followed by a HPLC-PDA technique. A good linearity was obtained with correlation coefficients in the range of 0.9908–0.9954 (Table 1).

#### 2.3.2. Accuracy and Precision

The accuracy of the method was validated by the spiked recovery test which is described below: Two concentration levels of the two analytes (100 and 200 ng mL^−1^) were added to a blank and samples separately, and then the resulting two sets of spiked samples were extracted and analyzed. The spiked recoveries were between 94.2% and 107.4% with RSD values from 2.8% to 6.5% (Table 2), which showed that the proposed method is accurate and reliable.

#### 2.3.3. Limits of Detection and Quantification

The LOD and LOQ values were calculated from the calibration curve obtained from model solutions [31,32] and the data are presented in Table 1. LOD and LOQ values were calculated at 3.3 and 10 σ/S, respectively, where σ is the standard deviation of the intercept and S is the slope of the regression line determined from the calibration curve.

#### 2.3.4. Selectivity

The selectivity of the method is the ability to distinguish between the target analytes and other substances or interferences in the real samples. In order to determine the selectivity, a sequence of blank tomato samples obtained from the local market was subjected to extraction and analysis. No interference peaks were observed within the retention time window for all the inspected samples.

#### 2.3.5. Robustness

The robustness of the developed method was examined by making small but deliberate variations in parameters such as flow rate of the mobile phase (1.10, 1.20, and 1.30 mL min^−1^), mobile phase composition in the isocratic elution (75% ACN, 70% ACN, 65% ACN, column temperature (35, 40, 45 °C), injection volume (8, 10, 12 µL), amount of magnetic sorbent (45, 50, 55 mg), and pH of the sample matrix (6.5, 7.0, 7.5). Insignificant differences in peak areas were observed with the RSD values in the range of 1.1–5.2 for all variables. The results suggested that these variations had little effect on the method performance, demonstrating the robustness of the method.

#### 2.3.6. Application in Real Sample

The newly developed MSPE coupled with the HPLC-PDA technique was applied to determine residues of two pesticides in environmental water samples collected from Sivas city, Turkey. The results showed in Table 2 that none of the studied pesticide was detected in samples.

#### 2.3.7. Performance Comparison between the Current and Other Reported Methods

The performance of the proposed method was compared with other sample pretreatment methods from the viewpoint of LOD, linear range, sample, and detection tool. As shown in Table 3, the newly developed MSPE-HPLC-PDA method has a good and useful linear range for FEN and PRO pesticides compared to other reported methods, demonstrating that the proposed method is sensitive, efficient, and repeatable. Spectrophotometric and GC based methods have problems about selectivity and sensitivity [13,33,34]. The proposed method has a wider linear range than most of methods in literature [6,14,16,19,35,36].

## 3. Materials and Methods

### 3.1. Reagents and Standard Solutions

All reagents used were of analytical grade. Ultra-pure water with a resistivity of 18.2 MΩ was used in all experiments and was provided by an ELGA (Flex III, Lane End, UK) water purification system. Fenitrothion and propoxur pesticide standard were purchased from Sigma-Aldrich (Steinheim, Germany). HPLC grade solvents, methanol, acetonitrile, and isopropyl alcohol were from Merck (Darmstadt, Germany). The stock solutions (200 mg/L) of pesticides were prepared by dissolving each of them in methanol. The working solutions were prepared by appropriate dilution of the stock solutions with double-distilled water. All the standard solutions were stored at 4 °C and brought to ambient temperature just prior to use.

A mixture stock solution containing propoxur and fenitrothion at 100.0 ng mL^−1^ was prepared in methanol. A series of standard solutions were prepared by mixing an appropriate amount of the stock solution with methanol in a 10 mL volumetric flask. 10 mM of Britton Robinson (BR) buffer (10 mM boric acid, 10 mM glacial acetic acid, and 10 mM phosphoric acid), with 100 mM NaCl was used in all the experiments at different pH values ranging from pH 2.0–10.0.

### 3.2. Instrumentation

The chromatographic system used was equipped with a pump model LC20-AD (Shimadzu, Tokyo, Japan), a thermostatic oven, CTO-10 AS (Shimadzu), auto sampler, SIL-20AC (Shimadzu), and a DAD detector model SPD-M20A (Shimadzu). An LC solution software (Shimadzu) was used to transfer data to the computer. A Luna Omega C18 (250 × 4.6 mm, 5 µm) column was used for chromatographic separation. A pH meter with a glass-calomel electrode (Selecta, Spain) was used to measure the pH values. An ultrasonic water bath (Kudos, China) was used for sample preparation.

Previous determinations, all solvents used in the chromatographic system were filtered through a 0.45 µm PDFA membrane filter (HNWP, Millipore, Burlington, MA, USA) using a vacuum pump (Buchi, Switzerland) and degassed for 10 min in an ultrasonic bath (JP Selecta, Barcelona, Spain).

Field-emission scanning electron microscope (Carl Zeiss-Gemini 500, Jena, Germany) was used to examine the morphology of decanoic acid modified magnetic nanoparticles and prove formation of magnetic nanoparticles by EDX analysis. The XRD measurements of decanoic acid modified magnetic nanoparticles was carried out by a Bruker AXS D8 advance X-ray powder diffractometer. FT-IR analysis for decanoic acid modified magnetic nanoparticles was continued by Perkin-Elmer Spectrum 400 FT-IR spectrometer (Waltham, MA, USA). TGA analysis was obtained with a Perkin Elmer-Diamond TGA analyzer. Thermo gravimetric analysis was performed in the range of 50–700 °C, N_2_ medium, and 10 °C/min^−1^ temperature rise rate.

### 3.3. HPLC-PDA Operating Conditions

**Figure 10:**
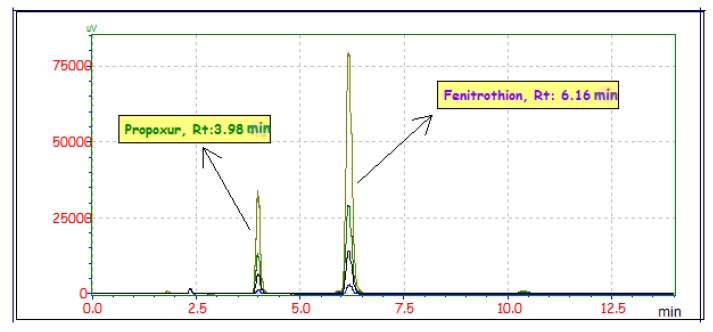
Chromatogram after magnetic solid-phase extraction (MSPE) for pesticide molecules.

The mobile phase composition was ACN:water (70:30) at an isocratic mode throughout the analysis. The flow rate was 1.2 mL min^−1^. The detector wavelength was operated at 269 and 271 nm for FEN and PRO, respectively. The column temperature was maintained at 30 °C and injection volume was 10 µL for all determinations. The HPLC system was optimized to obtain better signal-to-noise ratio of pesticides in a single chromatographic analysis, the best peak shape, an appropriate run-time, and better peak resolution. The column was flushed with 50% methanol to completely elute the remaining pesticides and the other organic compounds for 60 min after each day’s work. Peaks in the chromatograms were identified by comparison with retention times and UV spectra of standards. The peak area was considered for quantification. The obtained chromatogram after MSPE by using model solutions including PRO and FEN pesticides was presented at four increasing levels in Figure 10. Absorption spectrums obtained from DAD detector were given as Supplementary 1.

### 3.4. Synthesis of Magnetic Nanoparticles

A well-known synthesis approach in literature was used for obtaining magnetic material [33,34]. Briefly, a mixture of 0.745 g of FeCl_3_.6H_2_O and 0.383 g of FeSO_4_.4H_2_O were solved in 50 mL of 3 M HCl during stirring at a speed of 600 rpm. Then, 100 mL of 50% ethanol was added to the solution and temperature was kept on at 85 °C on a magnetic stirrer. The synthesis reaction was started by dropping 20 mL of ammonia throughout 10 min in inert medium of nitrogen gas. The obtained magnetite Fe_3_O_4_ particles were collected from the black solution by the help of external magnet, washed three times by using 50% MeOH, and dried in the oven at 60 °C for 6 h.

In the second step; 1 g Fe_3_O_4_ particles was dispersed in 80% ethyl alcohol solution including 1 mL of ammonia. The mixture was stirred at 600 rpm and 80 °C for 1 h after 1 mL of tetraethyl orthosilicate (TEOS) were added to the solution. Then, 200 mg decanoic acid was solved in 2 mL ethanol and added to the solution on a magnetic stirrer. The mixture was stirred for 6 h. The magnetic particles were removed from the solution by an external magnet and washed five times by 50% ethanol and then allowed to dry.

### 3.5. The Proposed Method of Magnetic Solid-Phase Extraction

50 mg of the synthesized magnetic material was weighed into a falcon tube and washed two times with 2 mL of ultrapure water. Then, 2 mL pH 7 buffer solution was added on the particles. Then, 20 mL sample solution including pesticides was added to the tubes and the volume of tubes was completed until 50 mL with ultrapure water. For adsorption of target molecules on magnetic particles surface, the tubes were placed on an orbital shaker at 80 rpm for 30 min. At the end of this period, the magnetic solid phase was easily separated by using a Neodymium magnet. After separation of aquatic phase, 400 µL isopropanol (2-propanol) was added to the tubes and they vortexed for 40 s in order to facilitate passing of pesticides molecules to organic phase. Then, the samples were filtered through a 0.45 μm syringe type filter and submitted to HPLC micro vials. After every use, the magnetic particle was washed with 1 mL isopropanol and 1 mL ethanol two times. The solid phase was ready for re-use after drying at the end of this procedure.

### 3.6. Preparation of Environmental Water Samples

The accuracy and applicability of the proposed method were tested with recovery tests by using various environmental water samples. Samples were prepared by using the methods in literature [37,38]. Briefly, natural samples from river, lake, and pond collected in Sivas, Turkey (Kızılırmak River, Tödürge Lake and 4 Eylül pond) were selected for analysis. After collection, the samples were immediately transported to the laboratory and stored in the dark. Prior to the analysis, water samples were filtered through a 0.45-μm PTFE filter and were stored at 4 °C. All samples were analyzed in triplicate using the developed MSPE procedure and HPLC-PDA system. Water samples were also used in this study to calculate the recovery of pesticides at two concentration levels (100 and 300 ng mL^−1^).

## 4. Conclusions

Novel magnetic particles with surface grafted decanoic acid were fabricated by the chemical bonding method and were employed as a new magnetic solid phase sorbent for propoxur and fenitrothion pesticides in environmental water samples. The developed decanoic acid modified magnetic particles could provide rapid separation of propoxur and fenitrothion from the complex environmental water samples with high selectivity and demonstrated high extraction efficiency for test targets in a short-time thanks to the large surface area. The proposed approach based on MSPE-HPLC-PDA displayed satisfactory performance and could be used as a good alternative to reported analytical methodologies for trace determination of PRO and FEN in real environmental water samples.

## Figures and Tables

**Table 1:** Analytical figures of merit of the proposed method.

Parameter	Before MSPE		After MSPE	
	Fenitrothion	Propoxur	Fenitrothion	Propoxur
Linear range	2.0–20.0 μg mL^−1^	2.0–20.0 μg mL^−1^	5.0–800.0 ng mL^−1^	10.0–800.0 ng mL^−1^
LOD	0.57 μg mL^−1^	0.57 μg mL^−1^	1.43 ng mL^−1^	3.15 ng mL^−1^
LOQ	1.88 μg mL^−1^	1.88 μg mL^−1^	4.93 ng mL^−1^	9.86 ng mL^−1^
RSD (%)	3.8	3.6	2.9	3.2
Slope	10.218	1.473	1348.8	173.76
(R^2^)	0.9972	0.9985	0.9954	0.9908
Preconcentration Factor ^a^	-	-	125	125
Enhancement Factor ^b^	-	-	132	118

^a^ Preconcentration factor is defined as the ratio of the initial solution volume (50 mL) to the volume of final solution (400 µL); ^b^ Enhancement factor is defined as ratio of slope of calibration before and after MPSE.

**Table 2:** Relative recovery values and reproducibility data for propoxur and fenitrothion pesticides in various environmental water samples after MSPE procedure (N:3).

Sample	Added ng mL^−1^	Found ^a^ ng mL^−1^		RSD %		Recovery %	
		Propoxur	Fenitrothion	Propoxur	Fenitrothion	Propoxur	Fenitrothion
River water I	-	<LOD	<LOD	-	-	-	-
	100.0	94.2 ± 2.7	103.2 ± 3.4	2.8	3.3	94.2	103.2
	200.0	206.1 ± 13.5	202.4 ± 8.4	6.5	4.1	103.5	101.2
Lake water II	-	<LOD	<LOD	-	-	-	-
	100.0	97.9 ± 3.8	99.5 ± 2.9	3.9	2.9	97.9	99.5
	200.0	192.1 ± 10.5	203.7 ± 9.8	5.5	4.8	96.1	101.8
Pond water	-	<LOD	<LOD	-	-	-	-
	100.0	107.4 ± 3.6	95.8 ± 3.9	3.4	4.0	107.4	95.8
	200.0	193.4 ± 8.9	205.8 ± 8.5	4.6	4.1	96.7	102.9

^a^ The average value of five replicates ± standard deviation.

**Table 3:** Comparison of the new method with other reported methods.

Preconcentration Method	Determination Method	Target Molecule	LOD	Linear Range	Applications	Reference
Quenchers based method	Gas chromatography-flame photometric detector	Fenitrothion	0.005 μg mL^−1^	0.005–5.0 μg mL^−1^	Tomatoes	[13]
Dispersive solid-phase microextraction	HPLC-UV	Fenitrothion	0.1 μg L^−1^	0.3–50.0 μg L^−1^	Water and fruit samples	[6]
Electrospun polystyrene nanofibers as solid-phase extraction sorbent	HPLC-DAD	Fenitrothion	0.07 ng mL^−1^	0.5–50.0 ng mL^−1^	Environmental waters	[14]
Magnetite octadecylsilane nanoparticles	HPLC-UV	Fenitrothion	0.014 ng mL^−1^	0.03–30 ng mL^−1^	Environmental water	[35]
Magnetic solid-phase extraction	Spectrophotometry	Fenitrothion	0.5 ng mL^−1^	2–230 ng mL^−1^	Environmental and biological samples	[34]
Magnetic solid-phase extraction	Gas chromatographic determination	Fenitrothion	3.9 ng mL^−1^	10–50000 ng mL^−1^	Fruit juices	[33]
Magnetic solid-phase extraction	HPLC-UV	Fenitrothion	0.2–0.8 μg L^−1^	1–100 μg L^−1^	Water samples	[16]
Magnetic solid-phase extraction	HPLC-UV	Propoxur	0.2 ng g^−1^	1.0 –100.0 ng g^−1^	Apple sample	[19]
Solid-phase extraction	HPLC-UV	Propoxur	0.05 ng g^−1^	0.2–80.0 ng g^−1^	Cucumber and watermelon samples	[36]
Magnetic solid-phase extraction	HPLC-PDA	Propoxur Fenitrothion	1.43 ng mL^−1^	5–800 ng mL^−1^	Environmental waters	This Study
			3.15 ng mL^−1^	10–800 ng mL^−1^		

## Supplementary Materials

The following are available online, Figure S1: Absorption spectrum of PRO and FEN obtained from DAD detector.
