# Supplementary figures and images for: Analytical Methodology for Trace Determination of Propoxur and Fenitrothion Pesticide Residues by Decanoic Acid Modified Magnetic Nanoparticles
Source: Molecules. 2019 Dec 17;24(24):4621. doi: 10.3390/molecules24244621 (PMC6943547; doi:10.3390/molecules24244621)

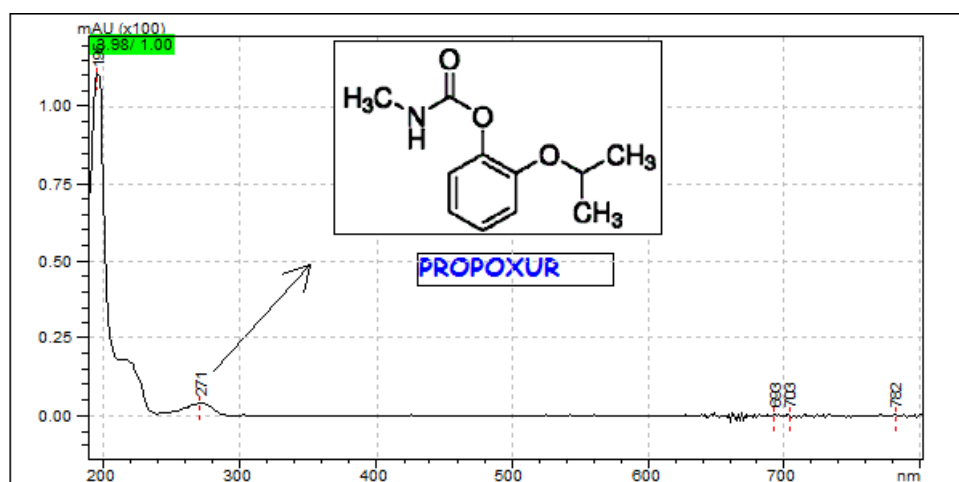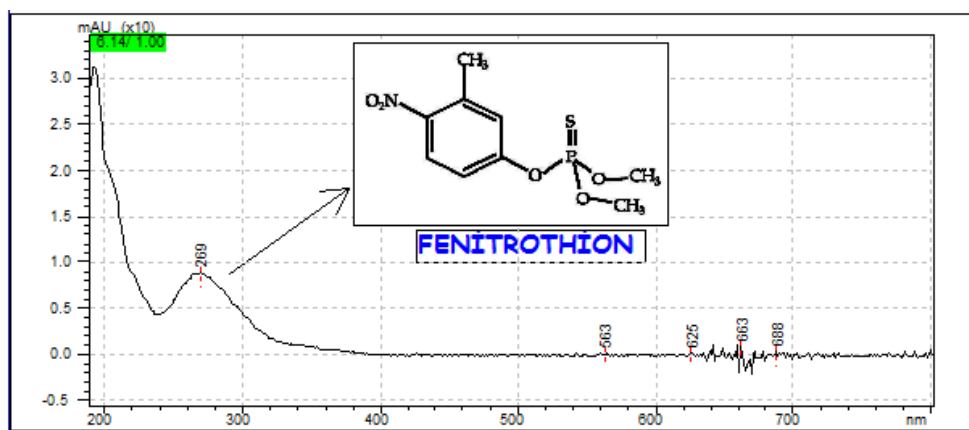

S1. Absorption spectrum of PRO and FEN obtained from DAD dedector

Supplement: Supplementary file 1 [file molecules-24-04621-s001.pdf]
